# The orthographic similarity structure of English words: Insights from network science

**DOI:** 10.1007/s41109-018-0068-1

**Published:** 2018-06-25

**Authors:** Cynthia S. Q. Siew

**Affiliations:** 1 Department of Psychology, University of Warwick, Coventry, CV4 7AL UK; 2 Department of Psychology, National University of Singapore, Singapore, Singapore

**Keywords:** Network science, Visual word recognition, Language network, Orthography, Speeded naming, Lexical decision, English lexicon project

## Abstract

Network science has been applied to study the structure of the mental lexicon, the part of long-term memory where all the words a person knows are stored. Here the tools of network science are used to study the organization of *orthographic* word-forms in the mental lexicon and how that might influence visual word recognition. An orthographic similarity network of the English language was constructed such that each node represented an English word, and undirected, unweighted edges were placed between words that differed by an edit distance of 1, a commonly used operationalization of orthographic similarity in psycholinguistics. The largest connected component of the orthographic language network had a small-world structure and a long-tailed degree distribution. Additional analyses were conducted using behavioral data obtained from a psycholinguistic database to determine if network science measures obtained from the orthographic language network could be used to predict how quickly and accurately people process written words. The present findings show that the structure of the mental lexicon influences lexical access in visual word recognition.

Within the cognitive sciences, the tools of network science have been applied to study the structure of the mental lexicon, the part of long-term memory where all the words a person knows is stored (Aitchison 2012). The mental lexicon can be viewed as a language network, where nodes represent words and edges represent relationships between words. Words can be related to other words in different ways—semantically (i.e., a word’s meaning; cat-dog), phonologically (i.e., the sounds of words; /k@t/−/h@t/), and orthographically (i.e., a word’s spelling; ‘cat’-‘cap’). Past work has shown that semantic (Steyvers and Tenenbaum 2005) and phonological (Vitevitch 2008) language networks have a small-world structure and that the structure of these networks influences various aspects of language processing—such as language acquisition (Hills et al. 2009) and spoken word recognition (Siew and Vitevitch 2016).

However, to date, not much is known about the orthographic language network, where edges in the network represent orthographic similarity relationships between words (i.e., whether words have similar *written* representations or spellings). Conceptualizing lexical representations as an orthographic network will build on previous psycholinguistic work demonstrating that orthographic similarity among words affects reading speeds and accuracies (Andrews 1997) by providing new ways of quantifying and investigating the orthographic similarity structure of language. In this paper, an orthographic network will be constructed for the English language and its overall network structure will be analyzed. To demonstrate the importance of applying network science approaches to the field of psycholinguistics and the cognitive sciences, additional analyses will be conducted to determine if the structure of the orthographic language network influences people’s visual word recognition performance.

Psycholinguists have long been interested in how words are organized and retrieved from the mental lexicon. One model was proposed by Murray and Forster (2004), where words in the mental lexicon were ordered based on the frequency of occurrence, allowing more common words to be retrieved more readily than less common words (i.e., the word frequency effect; Brysbaert and New 2009). Other models have emerged to account for the cognitive processes involved in reading and visual word recognition—these include interactive-activation models (McClelland and Rumelhart 1981; Morton 1969), dual-route models (Max Coltheart et al. 2001) and connectionist models (Seidenberg and McClelland 1989).

Network science can provide psycholinguists with another way of representing the organization of lexical representations within the mental lexicon. In a phonological language network, nodes represent phonological representations and connections are placed between words that are phonologically similar to each other (Vitevitch 2008). The structure of the phonological language network has been shown to influence spoken word recognition in a variety of psycholinguistic tasks (Siew 2017; Siew and Vitevitch 2016; Vitevitch et al. 2014). In a semantic language network, connections are placed between words that share semantic features or co-occur in language corpora (Steyvers and Tenenbaum 2005). The structure of the semantic language network has been shown to influence various language-related processes such as language acquisition in typically developing (Hills et al. 2010) and non-typically developing children (Beckage et al. 2011), as well as a variety of other cognitive processes related to semantic representation (De Deyne et al. 2016), creativity (Kenett et al. 2016), and human learning (Karuza et al. 2016). Finally, the syntactic dependency structure of language can also be represented as a network, leading to new insights into linguistic theories and language acquisition (Corominas-Murtra et al. 2009; Liu 2008; Solé et al. 2010). For a review detailing how network science has been applied more broadly in the cognitive sciences, see Baronchelli et al. (2013).

Previous psycholinguistic work has demonstrated that orthographic similarity among words affects reading speeds and accuracies. In a review of the literature surrounding orthographic neighborhood effects, Andrews (1997; see also Grainger 1992) concluded that words with more orthographic neighbors (i.e., words that are similarly spelled to the target word) were more efficiently processed (i.e., a facilitatory effect), although others have argued that orthographic neighbors play an inhibitory role in lexical access (Perea and Rosa 2000; Davis et al. 2009). This is a central research question in the field because it can lead to insights regarding the processes underlying visual word recognition. For instance, a key feature of interactive-activation models (e.g., McClelland and Rumelhart 1981; Grainger and Jacobs 1996) is that lexical access is the outcome of competitive processes among partially activated word candidates, which suggest that increased orthographic similarity among words should inhibit lexical access—a notion that is inconsistent with prior work showing a facilitatory effect of orthographic neighbors (Siakaluk et al. 2002).

In previous work, however, the operationalization of orthographic similarity was largely based on the local structure of words (i.e., number of same-length neighbors), although there have been some attempts to redefine the operationalization of orthographic similarity to include addition and deletion of letters (Davis et al. 2009), or one based on the mean edit distance of a word’s 20 closest orthographic neighbors (Yarkoni et al. 2008). The tools of network science could be used to provide new ways of quantifying orthographic similarity at both the local and global levels of the language network.

Prior work by Iyengar and colleagues suggested that the overall orthographic structure of language could have implications for navigating the mental lexicon. In Iyengar et al. (2012), participants played a “word-morph” game where they had to find a sequence of words such that the first word could be transformed to the second word (of the same length) by changing a single letter. For example, the sequence of words to get from “try” to “pot” was “try-toy-ton-tot-pot”. The results indicated participants were much faster at the game when they learned to make use of “landmark” words to find the correct sequence of words. These landmark words were in fact nodes in the orthographic network of three-letter English words that had high closeness centrality—a network science measure indicating the inverse of the sum of distances of a node to all other nodes in the network (Borgatti and Everett 2006). High closeness centrality words were “close” to many other words in the network. Iyengar et al.’s findings suggest that the network structure of *orthographic* word forms (albeit one that contained only three-letter words) has behavioral consequences as one navigates the mental lexicon and there could be similar implications for lexical retrieval. While the results from Iyengar et al. provide some initial evidence that the orthographic structure of language can influence lexical processes, there were two limitations: (i) only words with three letters were considered and (ii) a somewhat non-traditional language task was used. Considering only words with three letters would have led to the exclusion of a large proportion of words in the language. In order to examine how lexical processes occur within a complex language structure, it is important to construct an orthographic network with words of various lengths, and make use of well-established experimental paradigms in psycholinguistics to investigate these lexical processes.

To address the first limitation, an orthographic language network was constructed using a larger set of words; specifically 40,468 English words (mean letter length = 7.99; *SD* = 2.46) that were obtained from the English Lexicon Project (ELP; Balota et al. 2007), a database containing lexical and behavioral data collected from thousands of participants. An undirected edge was placed between two words that differed by a Levenshtein edit distance of 1 (i.e., whether the first word could be transformed into the second via the substitution, addition, or deletion of one letter). For instance, the word ‘cat’ would be connected to ‘hat’, ‘chat’, and ‘at’ (see Fig. 1 for the ego network of the word ‘cat’). This is consistent with the way that Vitevitch (2008) constructed the phonological language network, where links were placed between pairs of words that differed based on the substitution, deletion, or addition of one phoneme in any position within the word—a well-established operationalization of phonological similarity (Luce and Pisoni 1998).

**Fig. 1:**
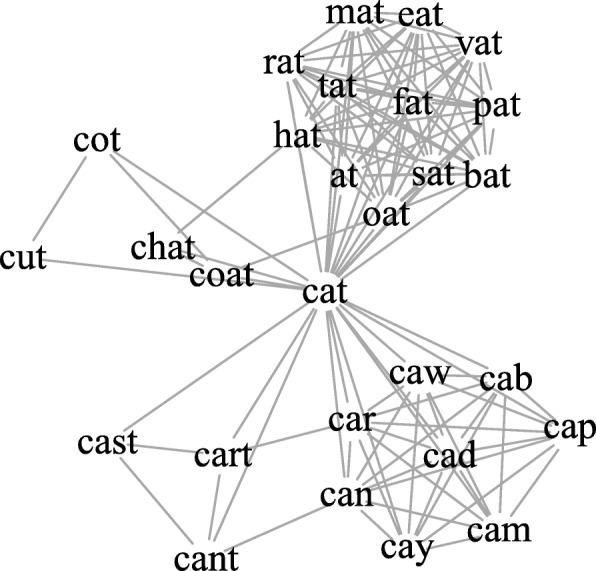
The orthographic structure of the ego network of the word ‘cat’. An undirected and unweighted edge was placed between two words that differed by a Levenshtein edit distance of 1 (i.e., whether the first word could be transformed into the second via the substitution, addition, or deletion of one letter)

To address the second limitation and to demonstrate the relevance of applying network science approaches to psycholinguistics, additional analyses will be conducted using behavioral data obtained from the English Lexicon Project to determine if network science measures obtained from the orthographic language network could be used to predict how quickly and accurately people process written words.

*Hypotheses*. Based on prior analyses of phonological language networks in various languages (Arbesman et al., 2010; Vitevitch 2008), one might hypothesize that the overall network structure of the orthographic network of English to also have similar characteristics. Vitevitch (2008) found that the phonological language network of English had a somewhat “small” large connected component consisting of about 33% of all nodes, and the large connected component had a small-world structure, with a small average path length and high average clustering coefficient relative to a comparable random network. It is hypothesized that the orthographic language network of English would show similar characteristics.

## Section 1: Constructing the orthographic network of English

### Method & Results

The orthographic network was constructed using 40,468 English words obtained from the English Lexicon Project (Balota et al. 2007). The English Lexicon Project represents a multi-institution collaboration to collect behavioral and descriptive data for over 40,000 English words. The behavioral data were collected from participants across six universities who completed lexical decision and speeded naming tasks, and descriptive data referred to various lexical measures for individual words, such as frequency counts based on various corpora. All data can be freely downloaded from this website: http://elexicon.wustl.edu/). An examination of the 40,468 words in the English Lexicon Project revealed that they could be derived from approximately 15,000 word families, a number that is very close to the 18,269 word families used by Brysbaert et al. (2016) in their crowdsourcing study examining the average vocabulary size of an average adult, suggesting that the words in the ELP could be viewed as an approximation of the words that an average, literate adult native speaker of American English is expected to know. An undirected edge was placed between two words that differed by a Levenshtein edit distance of 1 (i.e., whether the first word could be transformed into the second via the substitution, addition, or deletion of one letter), such that the word ‘cat’ would be connected to ‘hat’, ‘chat’, and ‘at’ (see Fig. 1 for an ego network of the word ‘cat’). Note that this definition of orthographic similarity differs slightly from what is typically used in the psycholinguistic literature. One of the most widely used measures of orthographic similarity is Coltheart’s *N*, which represents the number of words that could be formed by only the *substitution* of a single letter (Coltheart et al. 1977). However, constructing the orthographic network (termed “Coltheart network”) using a substitution only measure led to a network that consisted of several small, fragmented components of words where each component consisted of words with the same lengths. To provide an indication of the sparseness of the Coltheart network, the largest connected component consisted of only 2468 words (~ 6% of the entire network) and the average degree was 1.29. On the other hand, using the ‘substitution-addition-deletion’ operationalization of orthographic similarity to construct the orthographic network (i) permitted the inclusion of words of varying lengths in the network and (ii) was consistent with the operationalization used to construct the phonological language network in Vitevitch (2008).

**Fig. 2:**
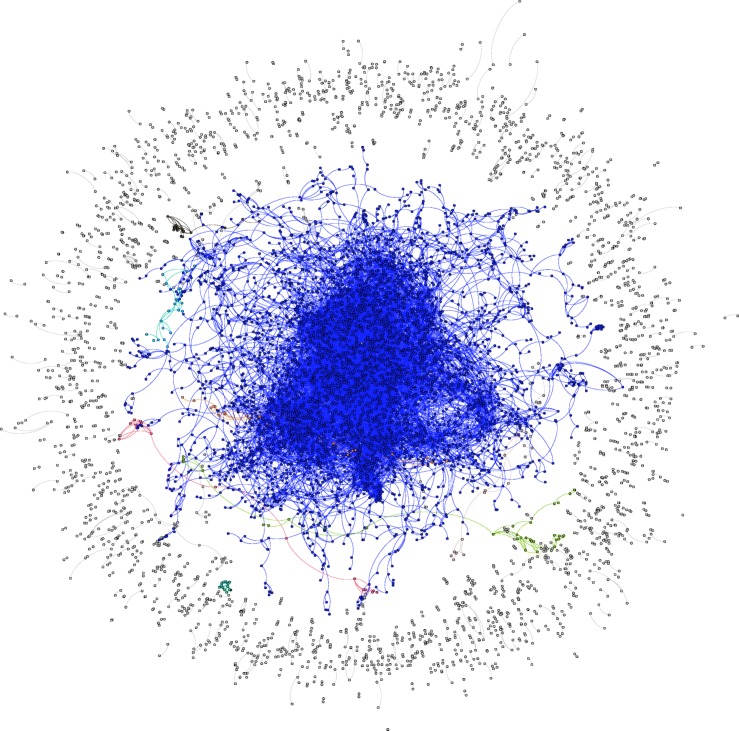
The overall orthographic network structure of the English language. Note that the largest connected component (nodes in blue) represented a somewhat limited proportion of the entire network, and the large numbers of smaller connected components and isolates (non-blue nodes)

The resulting orthographic language network consisted of 40,468 nodes and 41,514 edges. The sparseness of the network was due to the large proportion of nodes that either did not connect to any other nodes (40.74%; number of hermits = 16,488) or found in smaller connected components (4881 islands with sizes ranging from 2 to 34; 31.17%; number of nodes in islands = 12,615). See Fig. 2 for a visualization of the overall structure of the orthographic language network.

The largest connected component (LCC) of the orthographic language network consisted of 11,365 nodes and 32,759 edges. The LCC had an average degree <*k* > of 5.766, mean local clustering coefficient of 0.273, average shortest path length of 8.78, and network diameter *D* of 31. The *poweRlaw* package in R (Gillespie 2014), an implementation of the techniques proposed by Clauset et al. (2009) to test for the existence of power-law distributions, was used to examine the degree distribution of the LCC. The degree distribution was better approximated by a power-law distribution with a somewhat low exponent of 1.74 (bootstrapped *SE* = 0.338), as compared to exponential, log-normal, or Poisson distributions. The exponent of 1.74 is low compared to prior work examining scaling laws in various cognitive phenomena such as language, memory, learning, and perception (see Kello et al. 2010 for an overview). For instance, in Steyvers and Tenenbaum (2005)’s analyses of semantic language networks, they found that the exponents of various semantic networks ranged from 3.01 to 3.19. Therefore, one should be cautious in interpreting the degree distribution of the orthographic network as approximating a true power law.

To provide a baseline comparison, 100 random networks with the same number of nodes and edges as the LCC were constructed such that all edges were randomly rewired (i.e., Erdös-Rényi configuration model). For these random networks, the mean average degree <*k* > was 5.766 (*SD* = 0), the mean of the average local clustering coefficient was 0.000495 (*SD* = 0.000106), the mean average shortest path length was 5.53 (*SD* = 0.00412), and the mean network diameter D was 10.51 (*SD* = 0.522). In addition, a different set of 100 random networks was constructed by sampling the same number of nodes with replacement from the LCC (Snijders and Borgatti 1999). For each bootstrapped sample, the orthographic network was constructed based on the same 1-edit distance metric and the same network statistics were computed such that a distribution of these network statistics were obtained for 100 artificial networks. For these bootstrapped networks, the mean average degree <*k* > was 5.76 (*SD* = 0.0877), the mean of the average local clustering coefficient was 0.206 (*SD* = 0.00433), the mean average shortest path length was 9.61 (*SD* = 0.270), and the mean network diameter D was 37.1 (*SD* = 3.87). Following Snijders and Borgatti (1999), an independent samples *t*-test was conducted to compare the distributions of the network measures (average path length, average clustering coefficient, diameter) obtained from the random configuration networks and the bootstrapped networks. All *t*-tests were statistically significant; path length: *t*(99) = − 473.49, *p* < .001; clustering coefficient: *t*(99) = − 151.16, *p* < .001; diameter: *t*(99) = − 68.06, *p* < .001.

A final baseline comparison was also conducted by constructing 5 random lexicons (each having the same size of the LCC). These random lexicons were generated by creating random words that had the same lengths and same overall letter distributions as the original LCC. An orthographic network was then constructed for each the random lexicons based on the 1-edit distance similarity measure. On average, the random lexicon networks were even more sparsely connected as compared to the empirical orthographic network. The mean of largest connected component consisted of 2791.6 words (*SD* = 29.11, ~ 7% of the entire network) and the average degree was 0.335 (*SD* = 0.0137). Overall, the LCC of the orthographic network appears to have a heavy-tailed degree distribution and has a small-world structure, as characterized by a small average path length and large average clustering coefficient relative to a comparably sized random network. The size of the LCC of the orthographic network is also larger as compared to networks generated from artificial lexicons that preserved word length and letter distributions. A summary of the network measures for the orthographic network and the various random baselines is provided in Table 1.

**Table 1:** Summary of network measures derived from the largest connected component of the orthographic network, and the means and standard deviations of network measures of various baseline networks for comparison

	Network measures					
Network	Nodes	Edges	Average degree	Average clustering coefficient	Average shortest path length	Diameter
LCC	11,365	32,759	5.766	0.273	8.78	31
Random configuration networks (*N* = 100)	11,365 (0)	32,759 (0)	5.766 (0)	0.000495 (0.000106)	5.53 (0.00412)	10.51 (0.522)
Bootstrapped LCCs of edit distance 1 (*N* = 100)	8863.78 (116.35)	30,560.78 (562.08)	5.76 (0.0877)	0.206 (0.00433)	9.61 (0.270)	37.1 (3.87)
Random word networks (*N* = 5)	2791.6 (29.11)	6207.4 (274.98)	0.335 (0.0137)	0.267 (0.00819)	6.45 (0.0864)	18.8 (0.837)

Network measures computed for the largest connected component (LCC) of each network

It is interesting to note that unlike most other real-world complex networks where almost all nodes are connected to each other in a single large component (e.g., the semantic network; Steyvers and Tenenbaum 2005), the largest connected component of the orthographic language network only constituted a somewhat smaller portion of the entire network (~ 30%). This proportion, however, is in line with what was observed for the phonological network analyzed by Vitevitch (2008; see also Stella and Brede (2015), who found similar results in a much larger phonological network), where the largest connected component constituted ~ 33% of the entire network. Previous work examining Zipf’s law of word frequencies (which states that word frequencies decays as a power law of its rank; Zipf 1935) and other statistical properties of language (Ferrer i Cancho and Solé 2003) may offer an explanation. Frequent words tend to be short words that also tend to have several phonological and orthographic neighbors in the language; on the other hand, infrequent words tend to be longer words with few or no neighbors (Frauenfelder et al. 1993). Given that a limited proportion of words in the language are short, frequent words and that these are the same words with high degree and connectivity with other nodes in the network, it is perhaps not too surprising that the largest connected component of the orthographic network is somewhat “small”. Relevant to present discussion are a series of computational analyses conducted on the phonological language network by Stella and Brede (2015), which point to alternate explanations for the observed size of the LCC. Specifically, Stella and Brede (2015) preserved word length distributions of the lexicon and found that the LCC in their randomized networks was in fact *smaller* than the empirical network as compared to random expectation—suggesting that other lexical properties and features (apart from length) may play a contributing role in the larger proportion of words in the LCC observed in real world language networks. Another possibility is that the largest connected component of the orthographic network represents the “kernel lexicon” (Ferrer i Cancho and Solé 2001), a subset of the lexicon that all speakers of a given language is said to have knowledge of in order to facilitate successful communication. The overall structure of language networks may reflect evolutionary pressures for language systems to transmit information with high fidelity while minimizing memory constraints on human cognition (i.e., principle of least effort; Zipf 1935).

Finally, it should be emphasized that these analyses were conducted on the orthographic language network where edges were placed between orthographic representations that differed by an edit distance of 1 letter (i.e., a single character in an orthographic string). Although there are many other ways of operationalizing orthographic similarity—for instance, by using higher order edit distances or variable string kernel methods that could have led to a more densely connected network, or by considering graphemic similarity (i.e., ‘sch-’, or ‘th-’) instead of character similarity—an edit distance of 1 was chosen in order to be consistent with prior psycholinguistic research that used a similar metric for studying similarity effects in both visual (Davis et al. 2009) and spoken word recognition (Luce and Pisoni 1998) and previous computational analyses of phonological language networks (Arbesman et al., 2010; Vitevitch 2008; Siew, 2013). Importantly, the network measures generated from the 1-edit distance network would have a straightforward interpretation and be more relevant and applicable to the field of psycholinguistics (as compared to measures generated from networks constructed with an unnecessarily complex operationalization of orthographic similarity).

## Section 2: Analysis of the English Lexicon Project

The availability of databases containing item-level behavioral data and lexical variables for a large set of words has afforded large-scale, megastudies of visual word recognition where psycholinguists re-analyze the behavioral data in the ELP to test new hypotheses or evaluate the importance of new variables relative to established variables (Balota et al. 2007; New et al. 2006; Yap and Balota 2009). The aim of the following regression analyses was to demonstrate the relevance of applying network science approaches to psycholinguistics and determine if network science measures obtained from the orthographic language network could be used to predict how quickly and accurately people process written words in two language-related tasks. Note that although it is possible to generate a very large number of network measures to include in the regression model, the current paper focuses on degree, clustering coefficient, and closeness centrality as these measures build on previous work done in visual and spoken word recognition and lend themselves to clear, straightforward implications for lexical processing. As discussed earlier, neighborhood similarity effects (i.e., degree) has been previously examined in visual (Coltheart et al. 1977) and spoken word recognition (Luce and Pisoni 1998). In spoken word recognition, the clustering coefficient of words in the phonological network have been shown to influence recognition of spoken words and memory processes (Vitevitch et al., 2012) and closeness centrality has been previously shown to have implications for processing and mental navigation (Goldstein and Vitevitch, 2017; Iyengar et al. 2012).

*Hypotheses*. Based on prior work in visual word recognition, one would hypothesize that degree (i.e., the number of words that are orthographically similar to the target word) facilitates lexical processing (i.e., faster and more accurate responses in the lexical decision and speeded naming tasks). However, it is unclear whether the clustering coefficient and closeness centrality measures might influence recognition and reading times and accuracies as this paper represents the first attempt to investigate if similarity measures derived from an orthographic network representation influence visual word recognition. Given previous investigations showing the influence of phonological clustering coefficient and closeness centrality on spoken word recognition (Chan & Vitevitch, 2009; Goldstein & Vitevitch, 2017), however, one might expect that the orthographic measures of clustering coefficient and closeness centrality to have some influence on visual word recognition as well.

## Method

### Database

The ELP was a large multi-institutional project where researchers collected reaction time and accuracy data for 40,481 words obtained from thousands of participants in multiple institutions in the United States (see Balota et al. 2007 and the ELP website: http://elexicon.wustl.edu/ for more details). The 40,481 words were the stimuli presented to participants in the speeded naming and lexical decision tasks—two commonly used psycholinguistic tasks in visual word recognition research used to investigate how quickly and accurately people process written words. In these tasks, each participant is typically seated in front of a computer connected to a response box containing a dedicated timing board to provide millisecond accuracy for the recording of response times. Experimental software is used to randomize and present the stimuli (i.e., letter strings) on the computer screen.

In the speeded naming task, participants were instructed to read the word shown to them on a computer screen aloud as quickly and accurately as possible. Reaction times were measured from the stimulus onset to the onset of the participant’s verbal response. Verbal responses were recorded for offline scoring of accuracy. In the lexical decision task, participants were instructed to decide, as quickly and accurately as possible, whether the presented item was a real English word or a nonword (i.e., a made up word like ‘POIL’ that does not exist in English). If the item was a word, participants pressed the button on the response box labeled ‘WORD’ with their right index finger. If the item was a nonword, participants pressed the button labeled ‘NONWORD’ with their left index finger. Reaction times were measured from stimulus onset of the participant’s button press.

### Materials

Degree, clustering coefficient, and closeness centrality of each individual node in the LCC of the orthographic network was obtained. Degree refers to the number of edges (i.e., orthographic neighbors of a given word). Clustering coefficient, *C*, represents the extent to which the orthographic neighbors of a given word are also orthographic neighbors of each other (i.e., the extent to which a node’s orthographic neighborhood is fully connected; Watts and Strogatz 1998). Note that it was not possible to compute a meaningful value for the clustering coefficient of words in the LCC that have only 1 orthographic neighbor (*C* is undefined for these words). For the purposes of the present analyses, an arbitrary value of 0 was assigned as the value of *C* for these words. Closeness centrality measures the inverse of the average number of links between a word and all other words in the language network (Freeman et al. 1979). Specifically, closeness centrality ranges from 0 to 1, such that nodes with values close to 0 indicate that a given word is “far” from other words in the language network (i.e., many links must be traversed to get from that node to other nodes in the network) and words with values close to 1 indicate that a given word is “close” to other words in the network (i.e., few links must be traversed to get from that node to other nodes in the network). The degree, clustering coefficient, and closeness centrality of words in the LCC represent structural information of words in the network and are included as the group of predictors known as “network variables” in the regression. Table 2 shows the correlations among the three network variables. In addition, number of letters, number of phonemes, number of syllables, log of word frequency were included as the group of predictors known as “lexical variables” in the regression. These lexical measures were obtained for each word from the ELP database.

**Table 2:** Correlations between the three network measures included in the regression: degree, clustering coefficient, and closeness centrality

	Degree	Clustering Coefficient
Clustering Coefficient	0.14***	
Closeness Centrality	0.68***	0.07***

*N* = 11,365. All correlations were statistically significant, *p* < .001***

## Results

Item-level regression analyses were conducted on the mean reaction times and accuracies for 11,358 words (i.e., words in the LCC) for speeded naming and visual lexical decision tasks that were obtained from the ELP. The dependent variables consisted of *z*-scored reaction times (RT) and accuracy rates (ACC), averaged across participants for each word, for both speeded naming and lexical decision tasks. *Z*-scored reaction times refer to the standardization of each participant’s raw reaction times via a *z*-score transformation. Although both raw and *z*-scored reaction times are available in the ELP, *z*-scored reaction times, instead of raw reaction times, were analyzed to reduce the likelihood that a single participant may disproportionately influence the item means (Balota et al. 2007), and to be consistent with the protocol established by previous megastudies in analyzing *z*-scored RTs instead of raw latencies (e.g., Brysbaert and New 2009; Yap and Balota 2009).

A two-step hierarchical regression was conducted with the following predictors: Lexical variables (number of letters, number of phonemes, number of syllables, log of word frequency) added in Step 1 and network variables (degree, clustering coefficient, closeness centrality) added in Step 2. Partitioning the regression analysis into two steps was done to determine if the network variables could account for additional variance over previously entered variables. In all models, the inclusion of network variables in Step 2 significantly improved model fit (see Table 3), indicating that network variables were able to account for a small but significant amount of additional variance, beyond that of traditional lexical variables. Table 3 below shows a summary of the regression models at Step 2.

**Table 3:** Summary of regression results for speeded naming and lexical decision

(i) Speeded naming	RT		ACC	
Predictors				
*Lexical variables*				
Number of letters	*b* = 0.00930	*t* = 2.48	*b* = 0.00893	*t* = 7.86
	*SE* = 0.00375	*p* = .013*	*SE* = 0.00114	*p* < .001***
Number of phonemes	*b* = 0.00601	*t* = 1.67	*b* = 0.00746	*t* = 6.83
	*SE* = 0.00361	*p* = .095^+^	*SE* = 0.00109	*p* < .001***
Number of syllables	*b* = 0.0550	*t* = 10.24	*b* = − 0.0251	*t* = − 15.41
	*SE* = 0.00537	*p* < .001***	*SE* = 0.00163	*p* < .001***
Log frequency	*b* = − 0.0487	*t* = − 44.83	*b* = 0.0133	*t* = 40.49
	*SE* = 0.00109	*p* < .001***	*SE* = 0.000329	*p* < .001***
*Network variables*				
Degree	*b* = −0.00762	*t* = − 11.89	*b* = 0.00154	*t* = 7.91
	*SE* = 0.000641	*p* < .001***	*SE* = 0.000194	*p* < .001***
Clustering coefficient	*b* = − 0.00668	*t* = − 0.758	*b* = 0.00427	*t* = 1.60
	*SE* = 0.00882	*p* = .45	*SE* = 0.00267	*p* = .11
Closeness centrality	*b* = 0.704	*t* = 2.65	*b* = − 0.386	*t* = − 4.80
	*SE* = 0.266	*p* = .008**	*SE* = 0.0805	*p* < .001***
	Δ*R*^2^ = .0099		Δ*R*^2^ = .0055	
	*F* (3, 11,350) = 49.6, *p* < .001		*F* (3, 11,350) = 24.4, *p* < .001	
(ii) Lexical decision				
*Lexical variables*				
Number of letters	*b* = −0.0491	*t* = − 12.72	*b* = 0.0494	*t* = 21.06
	*SE* = 0.00386	*p* < .001***	*SE* = 0.00235	*p* < .001***
Number of phonemes	*b* = − 0.0169	*t* = − 4.56	*b* = 0.0176	*t* = 7.79
	*SE* = 0.00372	*p* < .001***	*SE* = 0.00226	*p* < .001
Number of syllables	*b* = 0.103	*t* = 18.55	*b* = − 0.0488	*t* = − 14.51
	*SE* = 0.00553	*p* < .001***	*SE* = 0.00336	*p* < .001***
Log frequency	*b* = − 0.0948	*t* = − 84.76	*b* = 0.0484	*t* = 71.22
	*SE* = 0.00112	*p* < .001***	*SE* = 0.000680	*p* < .001***
*Network variables*				
Degree	*b* = −0.00518	*t* = − 7.85	*b* = 0.00272	*t* = 6.78
	*SE* = 0.000661	*p* < .001***	*SE* = 0.000401	*p* < .001***
Clustering coefficient	*b* = 0.0185	*t* = 2.04	*b* = − 0.0104	*t* = − 1.88
	*SE* = 0.00909	*p* = .042*	*SE* = 0.00552	*p* = .059^+^
Closeness centrality	*b* = − 1.40	*t* = − 5.11	*b* = 0.288	*t* = 1.73
	*SE* = 0.274	*p* < .001***	*SE* = 0.166	*p* = .083^+^
	Δ*R*^2^ = .0066		Δ*R*^2^ = .0039	
	*F* (3, 11,350) = 45.1, *p* < .001***		*F* (3, 11,350) = 22.4, *p* < .001***	

^+^indicates p < .10, * indicates p < .05, ** indicates p < .01, *** indicates p < .001

### Speeded naming

#### Reaction times

The variables entered at Step 1 explained 25.7% of the variance in naming RTs, accounting for a significant proportion of the variance in naming RTs, *R*^*2*^ = .257, *F* (4, 11,353) = 982.8, *p* < .001. In Step 2, degree significantly predicted naming RTs, standardized *β* = − 0.00762, *t* = − 11.89, *p* < .001, such that words with high degree were more quickly named as compared to words with low degree. Closeness centrality significantly predicted naming RTs, standardized *β* = 0.704, *t* = 2.65, *p* = .008, such that words with high closeness centralities were more slowly named as compared to words with low closeness centralities. The influence of network variables accounted for an additional 0.99% of the variance, Δ*R*^*2*^ = .0099, *F* (3, 11,350) = 49.6, *p* < .001. Together, the variables entered at both steps explained 26.7% of the variance in naming RTs, accounting for a significant proportion of variance in naming RTs, *R*^*2*^ = .267, *F* (7, 11,350) = 590.1, *p* < .001.

#### Accuracies

The variables entered at Step 1 explained 15.4% of the variance in naming accuracies, accounting for a significant proportion of the variance in naming accuracies, *R*^*2*^ = .154, *F* (4, 11,353) = 516.8, *p* < .001. In Step 2, degree significantly predicted naming accuracies, standardized *β* = 0.00154, *t* = 7.91, *p* < .001, such that words with high degree were more accurately named as compared to words with low degree. Closeness centrality significantly predicted naming accuracies, standardized *β* = − 0.386, *t* = − 4.80, *p* < .001, such that words with high closeness centralities were less accurately named as compared to words with low closeness centralities. The influence of network variables accounted for an additional 0.55% of the variance, Δ*R*^*2*^ = .0055, *F* (3, 11,350) = 24.4, *p* < .001. Together, the variables entered at both steps explained 16.0% of the variance in naming accuracies, accounting for a significant proportion of variance in naming RTs, *R*^*2*^ = .160, *F* (7, 11,350) = 307.6, *p* < .001.

### Lexical decision

#### Reaction times

The variables entered at Step 1 explained 43.7% of the variance in lexical decision RTs, accounting for a significant proportion of the variance in lexical decision RTs, *R*^*2*^ = .437, *F* (4, 11,353) = 2201, *p* < .001. In Step 2, degree significantly predicted lexical decision RTs, standardized *β* = − 0.00518, *t* = − 7.85, *p* < .001, such that words with high degree were more quickly recognized as compared to words with low degree. Clustering coefficient significantly predicted lexical decision RTs, standardized *β* = 0.0185, *t* = 2.04, *p* = .042, such that words with high *C*s were less quickly recognized as compared to words with low *C*s. Closeness centrality significantly predicted lexical decision RTs, standardized *β* = − 1.40, *t* = − 5.11, *p* < .001, such that words with high closeness centralities were more quickly named as compared to words with low closeness centralities. The influence of network variables accounted for an additional 0.66% of the variance, Δ*R*^*2*^ = .0066, *F* (3, 11,350) = 45.1, *p* < .001. Together, the variables entered at both steps explained 44.3% of the variance in naming RTs, accounting for a significant proportion of variance in naming RTs, *R*^*2*^ = .443, *F* (7, 11,350) = 1292, *p* < .001.

#### Accuracies

The variables entered at Step 1 explained 33.9% of the variance in lexical decision accuracies, accounting for a significant proportion of the variance in lexical decision accuracies, *R*^*2*^ = .339, *F* (4, 11,353) = 1454, *p* < .001. In Step 2, degree significantly predicted lexical decision accuracies, standardized *β* = 0.00282, *t* = 6.78, *p* < .001, such that words with high degree were more accurately recognized as compared to words with low degree. The influence of network variables accounted for an additional 0.39% of the variance, Δ*R*^*2*^ = .0039, *F* (3, 11,350) = 22.4, *p* < .001. Together, the variables entered at both steps explained 34.3% of the variance in naming RTs, accounting for a significant proportion of variance in naming RTs, *R*^*2*^ = .343, *F* (7, 11,350) = 845.1, *p* < .001.

## General Discussion

In Section 1, an analysis of the orthographic forms obtained from a large database revealed that the LCC of the orthographic language network consisted of a small-world structure with a long-tailed degree distribution. In Section 2, regression analyses conducted on behavioral data from the ELP further showed that various network characteristics of words significantly predicted performance on speeded naming and lexical decision.

### Structure of orthographic network influences word recognition

Two key findings from the regression analyses (Section 2) will be highlighted. First, degree was a significant predictor of naming and lexical decision performance. High degree words were processed more quickly and accurately than low degree words—consistent with previous psycholinguistic work showing a processing advantage for words with many orthographic neighbors (albeit using slightly different operationalizations of orthographic similarity; Coltheart et al. 1977). Second, closeness centrality was a significant predictor of naming and lexical decision performance. High closeness centrality words were processed more slowly and less accurately than low closeness centrality words in naming, whereas high closeness centrality words were processed more quickly than low closeness centrality words in lexical decision. In lexical decision, words that are “close” to many words may appear to be more “word-like”, such that participants take a shorter time to decide if a letter string is a word. This is consistent with prior psycholinguistic work demonstrating that participants are faster to respond to more “word-like” words (Ratcliff et al. 2004). On the other hand, in the naming task, high closeness centrality words, being “close” to many other words in the lexicon, may experience greater competition from these words such that it worsens performance in the naming task where one has to retrieve the orthographic representation of a specific word from long-term memory.

One striking observation from the analyses is that the effect of degree and closeness centrality was in the opposite direction for naming whereas the effect of degree and closeness was in the same direction for lexical decision, This is especially interesting because degree and closeness centrality tend to be positively correlated with each other (see Table 2); however, in the naming task high degree facilitated performance whereas high closeness centrality hindered performance. This suggests that the orthographic similarity structure may operate differently at local and global levels of the system, and that the interaction of these local and global similarity effects may crucially depend on the task used to examine lexical processing.

### Implications for theories of word recognition

The outcome of these analyses have important theoretical implications for leading models of visual word recognition, which can be broadly classified into two groups: Dual route models and connectionist models. Dual route models posit the presence of two distinct, independent pathways in visual word recognition: One where meaning can be directly retrieved from the printed word, and one where grapheme-phoneme conversion rules are first applied to retrieve the word’s phonological representation before meaning is accessed (e.g., Coltheart et al. (2001)’s Dual Route Cascaded model of visual word recognition and production). Connectionist models (e.g., Seidenberg and McClelland (1989)’s Parallel Distributed Processing model) consist of orthographic units, phonological units, and a set of hidden units that interface between the orthographic and phonological units. Despite having very different architectural principles and modeling assumptions, both models have been successful at simulating and explaining a number of lexical effects in visual word recognition.

Interestingly, none of these models would predict any of the network measures derived from the orthographic language network to have an effect on word recognition because the models tend to focus on the cognitive *processes* that lead to successful lexical retrieval, and do not take into account how the overall similarity *structure* of orthographic word-forms within the mental lexicon affects lexical access. For instance, it is unclear as to how these models of visual word recognition would account for the closeness centrality effects found in the regression analysis, given that none of these models explicitly considered how lexical mechanisms operate within the complex language structure that exists in the mental lexicon. This represents an especially strong constraint that computational modelers of visual word recognition models should take into account, especially given the increasing amount of research showing that the structure of various cognitive networks constrains the types of cognitive processes that operate within these networks (Kenett, Levi, Anaki, & Faust, 2017; Vitevitch, Chan, & Roodenrys, 2012). For instance, Kenett, Levi, Anaki, and Faust (2017) recently showed that path length between words in a semantic network affected behavioral performance in a semantic relatedness judgment task and recall memory. This study demonstrates that cognitive processes such as spreading activation should be investigated in tandem with the underlying structure of the cognitive landscape that it operates on. Similarly, psycholinguists should also consider how the structure of the mental lexicon might constrain or inform the cognitive processes associated with lexical retrieval and word recognition.

As discussed in the Introduction, the most commonly used measure of orthographic similarity in visual word recognition is Coltheart’s *N* (Coltheart et al. 1977), which counts the *number* of neighbors (i.e., the degree of a node), but does not consider the *internal neighborhood structure* of these neighbors. The present findings of (i) an effect of orthographic clustering coefficient (a measure that quantifies the internal structure of a lexical neighborhood) and (ii) an effect of closeness centrality (a measure that quantifies a word’s overall structural importance in the network) on visual word recognition are theoretically important and should compel theories of word recognition to consider how the *structure* of the mental lexicon affects the cognitive processes that underlie word recognition.

### Future directions

This paper focused on the orthographic network of the English language; however a similar analysis can be conducted for the orthographic forms of other languages in order to determine if the overall network structure is similar to that of the English language, and examine if the network structure of these words also influence visual word recognition. Another possible future direction is to apply more advanced techniques from network science to analyze the overall structure of language as a multiplex network, where different layers in the multiplex represent different types of relations between words (e.g., semantic or phonological relationships). Indeed, there has been recent work focusing on representing the semantic and phonological relationships between words as a multiplex structure to explain language development and acquisition in children (Stella et al. 2017; Stella et al. 2018), and to account for language deficits in aphasic patients (Castro and Stella 2018). Incorporating orthographic information as another layer in the language multiplex could allow language scientists to better study the interrelationship between the orthography and phonology of words, especially in languages with a less transparent orthographic script (e.g., English and French; Katz and Frost 1992), and could have implications for understanding impaired reading processes in dyslexia or improve literacy training programs for children learning to read.

## Abbreviations

ACC: Accuracy
C: Clustering coefficient
ELP: English Lexicon Project
LCC: Largest connected component
RT: Reaction time
